# Association between Usual Sodium and Potassium Intake and Blood Pressure and Hypertension among U.S. Adults: NHANES 2005–2010

**DOI:** 10.1371/journal.pone.0075289

**Published:** 2013-10-10

**Authors:** Zefeng Zhang, Mary E. Cogswell, Cathleen Gillespie, Jing Fang, Fleetwood Loustalot, Shifan Dai, Alicia L. Carriquiry, Elena V. Kuklina, Yuling Hong, Robert Merritt, Quanhe Yang

**Affiliations:** 1 Division for Heart Disease and Stroke Prevention, Centers for Disease Control and Prevention, Atlanta, Georgia, United States of America; 2 Department of Statistics, Iowa State University, Ames, Iowa, United States of America; FuWai hospital, Chinese Academy of Medical Sciences, China

## Abstract

**Objectives:**

Studies indicate high sodium and low potassium intake can increase blood pressure suggesting the ratio of sodium-to-potassium may be informative. Yet, limited studies examine the association of the sodium-to-potassium ratio with blood pressure and hypertension.

**Methods:**

We analyzed data on 10,563 participants aged ≥20 years in the 2005–2010 National Health and Nutrition Examination Survey who were neither taking anti-hypertensive medication nor on a low sodium diet. We used measurement error models to estimate usual intakes, multivariable linear regression to assess their associations with blood pressure, and logistic regression to assess their associations with hypertension.

**Results:**

The average usual intakes of sodium, potassium and sodium-to-potassium ratio were 3,569 mg/d, 2,745 mg/d, and 1.41, respectively. All three measures were significantly associated with systolic blood pressure, with an increase of 1.04 mmHg (95% CI, 0.27–1.82) and a decrease of 1.24 mmHg (95% CI, 0.31–2.70) per 1,000 mg/d increase in sodium or potassium intake, respectively, and an increase of 1.05 mmHg (95% CI, 0.12–1.98) per 0.5 unit increase in sodium-to-potassium ratio. The adjusted odds ratios for hypertension were 1.40 (95% CI, 1.07–1.83), 0.72 (95% CI, 0.53–0.97) and 1.30 (95% CI, 1.05–1.61), respectively, comparing the highest and lowest quartiles of usual intake of sodium, potassium or sodium-to-potassium ratio.

**Conclusions:**

Our results provide population-based evidence that concurrent higher sodium and lower potassium consumption are associated with hypertension.

## Introduction

High blood pressure or hypertension is the leading preventable risk factor for cardiovascular diseases and is estimated to account for about 54% of deaths from stroke and 47% of deaths from coronary heart disease in adults worldwide [1]. Furthermore, the risk for cardiovascular disease increases progressively with blood pressure, beginning at pressures as low as 115/75 mmHg [2]. According to recent estimates, roughly one third of U.S. adults have high blood pressure [3], and in 2010, high blood pressure was estimated to be responsible for $156 billion in direct and indirect costs [4]. Results of randomized controlled trials have consistently shown reducing sodium intake reduces average blood pressure [5]–[7]. Conversely, observational studies indicate dietary potassium intake is negatively associated with blood pressure in some studies [8]–[10]; but not others [11]–[14].

Although the exact mechanisms by which sodium and potassium levels affect blood pressure are not well understood, evidence suggest that the altered sodium and potassium homeostasis play a key role in pathogenesis of hypertension [15]. However, population-based studies of the association between blood pressure or hypertension with the sodium-to-potassium ratio are limited by the use of intake estimates based on one 24-hour dietary recall [16], [17]. The lack of adjustment for day-to-day variability increases measurement error and may result in the estimates of the associations being biased toward the null. Furthermore, previous studies did not adjust for diabetes, heavy alcohol use, or physical activity, nor examined potential interactions between intake and age, sex, race/ethnicity or other covariates [16], [17]. In this study, we used 2005–2010 data from the National Health and Nutrition Examination Survey (NHANES) to estimate the associations of blood pressure and hypertension with sodium and potassium intake and their ratio among U.S. adults adjusting for within-person variability in intake. We additionally adjusted for potential confounding variables and tested for interactions between estimated intakes and age, sex, race/ethnicity, and other characteristics.

## Subjects and Methods

### Data Source

Our data source, NHANES, uses a complex, stratified, multistage probability cluster sampling design to collect health and nutritional data from a representative sample of the non-institutionalized U.S. population. The design and operation of NHANES was described previously [18]–[20]. Of 15,702 NHANES participants during 2005–2010 who were ≥20 years of age and with reliable information on the 1^st^ 24-hour dietary recall, we sequentially excluded participants who were pregnant (n = 439), those with missing blood pressure data (n = 594), those who did not report their diagnosed hypertension status (n = 29), those who reported being on a low sodium diet (n = 297), and those who reported taking antihypertensive medication (n = 3,780), yielding a sample of 10,563 participants for our analyses. Study protocols for NHANES were approved by the National Center for Health Statistics ethnics review board. Signed informed consent was obtained from all participants.

### Outcomes

The two main outcomes in this study were mean blood pressure and hypertension status. The reported blood pressure was the average of up to 3 readings obtained under standard conditions during a single physical examination [18]–[20]. Blood pressure was measured in a seated position after at least 5 minutes of rest using a mercury sphygmomanometer according to the standardized protocols for blood pressure measurements of the American Heart Association [21]. Participants' hypertension status was based on a combination of their self-reported history of any diagnosis of hypertension and on the average of the blood pressure readings taken during their NHANES examination. Participants were classified as having diagnosed hypertension if they indicated that a health care provider ever told them they had high blood pressure and as having undiagnosed hypertension if they indicated they had not been told they had high blood pressure but were found to have a mean systolic blood pressure ≥140 mmHg or a mean diastolic blood pressure ≥90 mmHg [22]. We excluded participants who took antihypertensive medications from our analysis because of the potential effect on the association between sodium and potassium intake and blood pressure.

### Covariates

Study covariates included race/ethnicity (non-Hispanic white, non-Hispanic black, Mexican-American, or others); body mass index (BMI); educational attainment (≤12 years or >12 years); smoking status (current smoker, former smoker, or never smoked); cardiovascular disease status (self-reported history of coronary heart disease, heart attack, angina, chronic heart failure, or stroke); diabetes based on participants' self-reported history of diabetes diagnosis, or use of insulin or other diabetic medications to lower blood glucose; chronic kidney disease status based on whether participants indicated they had “weak/failing kidneys”; and heavy user of alcohol, defined as self-reported consumption of more than two beverages per day for men and more than one beverage per day for women (heavy user or not heavy user) [23]. Due to changes in physical activity questions, the *2008 Physical Activity Guidelines for Americans* recommendation for all adults to avoid inactivity was utilized as a basic assessment of activity [24]. Physical activity status was based on whether participants reported engaging in at least 10 minutes of moderate- and/or vigorous-intensity activity per week (active or inactive).

### Estimating Sodium and Potassium Intake

Data on dietary sodium and potassium intake were assessed using two 24-hour dietary recalls. The first recall was administered in person, followed by a second recall administered via phone 3–10 days later. Nutrient values were assigned to foods using the U.S. Department of Agriculture (USDA) Food and Nutrient Database for Diet Studies (FNDDS) [25]. Trained interviewers administered the 24-hour dietary recalls using the automated multi-pass method. Sodium values of selected foods in the FNDDS were adjusted downward when participants reported using salt occasionally or less often during food preparation. Sodium from table salt was categorized as never/rarely, sometimes and often. No adjustment was made for consumption of potassium salts. Sodium and potassium from supplements and antacids were not included in the estimates of total intake.

### Statistical Analyses

To estimate the usual intake of sodium, potassium and their ratio accounting for between- and within-person variation in intake [26], we used PC-SIDE software (Software for Intake Distribution Estimation for the Windows OS) (Iowa State University, Version 1.0). The estimate of usual intake was adjusted for age in years, sex, race/ethnicity, first or second day of dietary recalls (all participants had first day and 87% had second day dietary recall), and the day of the week of the recall. The standard errors for mean usual intake were estimated using a set of Jackknife replication weights based on the first-day dietary sampling weights. For the association study, we estimated the best linear unbiased predictor of usual sodium and potassium intake and their ratio for each stratum of age (20–50 years vs. ≥51 years), sex, and race/ethnicity, a total of 16 strata [27].

We used multivariable linear regression to examine the association between participants' usual intake of sodium and potassium (per 1,000 mg/d) and their ratio (per 0.5 unit) and systolic and diastolic blood pressure. Because all three variables had an approximately linear relationship to blood pressure, we calculated the 12.5^th^, 37.5^th^, 62.5^th^, and 87.5^th^ percentiles distribution of the estimated usual intakes. Using the parameters from the linear regression models, we estimated the adjusted mean systolic and diastolic blood pressure of the 12.5^th^, 37.5^th^, 62.5^th^, and 87.5^th^ percentiles distribution of the estimated usual intakes. These adjusted means can be interpreted as the middle value of each quartile (Q1, Q2, Q3, and Q4) [28].

For risk of hypertension, we used multivariable logistic regression analysis to estimate adjusted odds ratio (OR) comparing Q4, Q3 and Q2 to the lowest quartile (Q1) using the similar approach as the linear regression models. For both linear and logistic regression models, covariates included were age as categorical variable (in every 5-year increment), gender, race/ethnicity, BMI, education, use of table salt, smoking status, history of cardiovascular disease, self-reported kidney disease, diabetes, alcohol use, and physical activity. We adjusted for sodium and potassium intake concurrently in the same model, and the models for sodium-to-potassium ratio estimates did not adjust for sodium or potassium intake.

We tested the interaction between the estimated intakes and covariates by including the interaction terms in the regression models and used Bonferroni adjustments for the multiple comparisons. Because of the significant interaction between age and potassium intake on systolic blood pressure, we presented the stratified results by age groups (20–29, 30–39, 40–49, 50–59, 60–69 and ≥70 years) by comparing the blood pressure at 10^th^ and 90^th^ percentiles of potassium intake within each age group. The differences in mean estimated intakes between hypertensive and non-hypertensive groups were tested using Z-tests based on the means and standard errors from PC-SIDE. SUDAAN version 9.3 was used to take into account for the complex sampling design. All tests were two-sided, and a p-value of <0.05 was considered statistically significant.

## Results

Compared to normotensive participants, hypertensive participants were older, more likely to be male, non-Hispanic black, former smokers, physically inactive, had higher BMI, and were more likely to have history of cardiovascular disease, diabetes and self-reported chronic kidney disease (Table 1).

**Table 1:** Comparison of selected characteristics between hypertensive and non-hypertensive participants among U.S. adults aged ≥20 years who were not taking antihypertensive medication, NHANES 2005–2010^1^^,^ 2.

Characteristics	Hypertension (n = 2178)	Normotension (n = 8385)	P value
**Age, years (mean±SE)**	50.3±0.57	41.1±0.29	<0.001
**Male**	56.4%	48.5%	<0.001
**Race/ethnicity**			
Non-Hispanic white	70.6%	69.8%	0.618
Non-Hispanic black	12.5%	9.7%	0.005
Mexican-American	7.7%	9.7%	0.014
Other	9.2%	10.9%	0.068
**Body mass index (mean±SE)**	29.3±0.26	27.4±0.13	<0.001
**History of cardiovascular disease** 3	7.9%	3.1%	<0.001
**Diabetes** 4	6.3%	3.5%	<0.001
**Self-reported chronic kidney disease** 5	1.9%	1.0%	0.002
**Smoking status**			
Current smoker	28.2%	24.9%	0.089
Former smoker	24.7%	20.9%	0.023
Never smoked	47.1%	54.2%	<0.001
**Heavy user of alcohol** 6	19.5%	17.1%	0.051
**Physically inactive** 7	47.2%	37.6%	<0.001

1 Sample size is unweighted.

Pregnant women and individuals missing data on blood pressure measurement and hypertension status, reporting being on a low sodium diet or taking antihypertensive medication are excluded.

2 Hypertension included both diagnosed and undiagnosed hypertension.

Participants were classified as having diagnosed hypertension if they indicated that a health care provider told them they had high blood pressure and as having undiagnosed hypertension if they indicated they had not been told they had high blood pressure but were found to have a mean systolic blood pressure ≥140 mmHg or a mean diastolic blood pressure ≥90 mmHg.

3 Cardiovascular diseases included self-reported history of coronary heart disease, heart attack, angina, chronic heart failure, or stroke.

4 Diabetes mellitus was based on participants' self-reported history of diabetes diagnosis, or use of insulin or other diabetic medications to lower blood glucose.

5 Chronic kidney disease status was based on whether participants indicated they had “weak/failing kidneys”.

6 Heavy user of alcohol was defined as self-reported consumption of more than two beverages per day for men and more than one beverage per day for women.

7 Adults were classified as physically inactive if participants reported engaging in less than 10 minutes of moderate and/or vigorous-intensity activity per week.

Overall, the study participants' average estimated usual intakes of sodium, potassium and sodium-to-potassium ratio were 3,569 mg/d, 2,745 mg/d, and 1.41, respectively, and did not differ by hypertension status (Table 2). However, among certain population subgroups, mean intakes did differ by hypertension status. Mean sodium intake was higher in hypertensive participants vs. those who were not among those participants aged 20–50 years or who were physically active. Mean sodium intake was lower in hypertensive participants vs. those who were not among women, among participants with a BMI<25.0, with diabetes, or who were physically inactive. Mean potassium intake was lower in hypertensive participants vs. those who were not among those participants who were aged ≥51 years or had diabetes, but mean potassium intake was higher in hypertensive participants among those with a BMI≥30, or who were physically active. The mean sodium-to-potassium ratio was higher among hypertensive participants vs. those who were not in both age strata, 20–50 years and ≥51 years (Table 2).

**Table 2:** Average usual intake of sodium, potassium and sodium-to-potassium ratio among U.S. adults aged ≥20 years who were not taking antihypertensive medication, by hypertension status and selected characteristics, NHANES 2005–2010.

Category^2^	Sample N		Usual sodium intake, mean (SE), mg/d^1^		Usual potassium intake, mean (SE), mg/d^1^		Sodium-to-potassium ratio, mean (SE)^1^	
	Hypertensive		Hypertensive		Hypertensive		Hypertensive	
	Yes	No	Yes	No	Yes	No	Yes	No
**All**	2178	8385	3561 (49.2)	3573 (30.5)	2770 (51.3)	2740 (24.1)	1.40 (0.02)	1.41 (0.01)
**Age, years**								
20–50	969	5998	3918 (88.4)	3684* (34.1)	2827* (67.9)	2701* (29.2)	1.54 (0.03)	1.48* (0.01)
≥51	1209	2387	3197 (71.2)	3251 (42.2)	2696 (50.3)	2846* (39.1)	1.27 (0.02)	1.21* (0.02)
**Sex**								
Male	1231	4202	4208 (71.0)	4232 (44.8)	3172 (59.9)	3148 (33.6)	1.42 (0.02)	1.45 (0.01)
Female	947	4183	2833 (54.8)	3018* (32.8)	2294 (57.5)	2388 (25.2)	1.39 (0.04)	1.37 (0.01)
**Race/ethnicity**								
Non-Hispanic white	1055	4003	3615 (69.5)	3620 (40.8)	2866 (56.4)	2821 (31.5)	1.37 (0.02)	1.38 (0.01)
Non-Hispanic black	470	1403	3355 (112.4)	3451 (64.1)	2340 (82.2)	2334 (42.2)	1.59 (0.04)	1.61 (0.02)
Mexican-American	396	1793	3077 (177.2)	3367 (64.9)	2638 (113.9)	2675 (38.3)	1.28 (0.05)	1.36 (0.02)
Other	257	1186	3815 (186.1)	3563 (64.2)	2701 (97.0)	2639 (44.6)	1.50 (0.06)	1.46 (0.02)
**Body mass index**								
<25.0	545	2945	3381 (104.0)	3541* (51.6)	2765 (100.4)	2751 (41.8)	1.36 (0.05)	1.38 (0.02)
25.0–29.9	744	2929	3522 (87.2)	3567 (45.5)	2802 (67.4)	2839 (30.5)	1.35 (0.03)	1.36 (0.02)
≥30.0	868	2466	3739 (81.7)	3633 (46.9)	2745 (64.8)	2602* (30.0)	1.47 (0.04)	1.50 (0.02)
**History of cardiovascular disease**								
Yes	212	334	3116 (146.1)	3219 (154.0)	2451 (124.7)	2577 (112.9)	1.43 (0.07)	1.35 (0.05)
No	1947	8028	3610 (54.5)	3585 (30.6)	2801 (54.1)	2744 (23.5)	1.40 (0.02)	1.41 (0.01)
**Diabetes**								
Yes	215	426	3052 (11.3)	3579* (138.2)	2421 (93.8)	2819* (95.1)	1.37 (0.05)	1.36 (0.04)
No	1962	7953	3596 (51.2)	3573 (30.9)	2793 (49.4)	2737 (24.2)	1.41 (0.02)	1.41 (0.01)
**Self-reported chronic kidney disease**								
Yes	63	97	2825 (271.7)	3147 (327.3)	2134 (176.2)	2417 (264.8)	1.45 (0.09)	1.49 (0.09)
No	2110	8276	3574 (50.3)	3577 (30.7)	2778 (52.1)	2742 (24.2)	1.40 (0.02)	1.41 (0.01)
**Smoking status**								
Current smoker	562	2056	3613 (102.0)	3643 (69.6)	2747 (75.3)	2690 (49.1)	1.45 (0.08)	1.50 (0.02)
Former smoker	553	1727	3726 (124.1)	3664 (66.9)	2950 (75.7)	2905 (48.0)	1.34 (0.03)	1.35 (0.03)
Never smoked	1061	4601	3428 (96.7)	3500 (39.1)	2680 (76.7)	2696 (31.4)	1.40 (0.03)	1.39 (0.01)
**Heavy user of alcohol**								
Yes	343	1244	3805 (123.7)	3659 (61.5)	2960 (107.3)	2792 (40.2)	1.40 (0.05)	1.40 (0.02)
No	1722	6663	3516 (67.7)	3573 (29.6)	2722 (55.9)	2737 (25.0)	1.41 (0.03)	1.41 (0.01)
**Physical activity**								
Active	985	4552	3822 (78.1)	3644* (37.4)	3006 (70.8)	2813* (28.7)	1.36 (0.02)	1.39 (0.01)
Inactive	1169	3790	3292 (66.3)	3457* (49.2)	2561 (44.4)	2620 (28.7)	1.44 (0.03)	1.43 (0.02)

1 A asterisk indicates p value<0.05 for the comparison between the hypertensive and non-hypertensive groups.

2 The exclusion criteria and definitions for covariates were the same as in Table 1.

**Figure 1:**
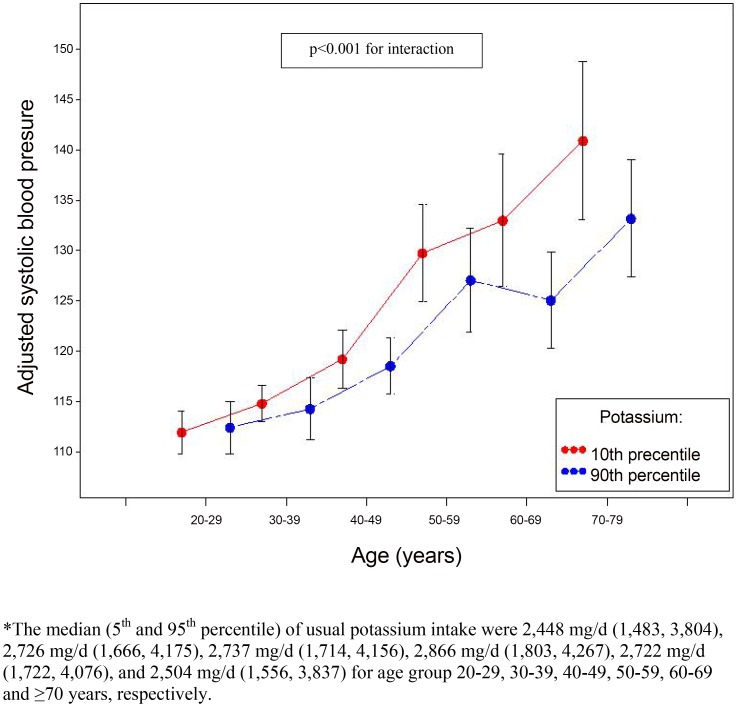
Adjusted systolic blood pressure (95% confidence interval) by 10^th^ and 90^th^ percentiles of potassium intake and age 20–29, 30–39, 40–49, 50–59, 60–69 and ≥70 years among adults aged ≥20 years who were not taking antihypertensive medication, NHANES 2005–2010.

After adjustment for potential confounders, intake of sodium, potassium and their ratio were significantly associated with systolic blood pressure, with an increase of 1.04 mmHg (95% confidence interval (CI), 0.27–1.82) and a decrease of 1.24 mmHg (95% CI, 0.31–2.70) for every 1,000 mg/d increase in sodium and potassium intake, respectively, and an increase of 1.05 mmHg (95% CI, 0.12–1.98) for every 0.5 unit increase in sodium-to-potassium ratio (Table 3). Overall, diastolic blood pressure was not associated with the estimated intakes, with one exception; every 1,000 mg/d increase of potassium intake was associated with 0.75 mmHg (95% CI, 0.22–1.28) decrease in diastolic blood pressure. The relationship between systolic blood pressure and potassium intake differed by age groups (p<0.001 for interaction). The effect of potassium intake on systolic blood pressure (comparing 90^th^ (3,751 mg/d) to 10^th^ percentile (1,831 mg/d)) appeared to be stronger among older age groups (Figure 1).

**Table 3:** Association between usual intake of sodium, potassium and their ratio and blood pressure among adults aged ≥20 years who were not taking antihypertensive medication, NHANES 2005–2010.

	Systolic		Diastolic	
Characteristics	β-coefficient^1^	p-value	β-coefficient^1^	p-value
**Usual sodium intake**				
Adjusted for age, sex and race/ethnicity only	1.48 (0.75–2.21)	<0.001	0.61 (0.05–1.18)	0.035
Fully-adjusted model^2^	1.04 (0.27–1.82)	0.001	0.36 (−0.17–0.90)	0.181
**Usual potassium intake**				
Adjusted for age, sex and race/ethnicity only	−1.87 (−2.7–1.03)	<0.001	−0.93 (−1.50–0.37)	0.002
Fully-adjusted model^2^	−1.24 (−2.70–0.31)	0.001	−0.75 (−1.28–0.22)	0.007
**Sodium-to-potassium ratio**				
Adjusted for age, sex and race/ethnicity only	1.66 (0.82–2.51)	<0.001	0.86 (0.21–1.50)	0.010
Fully-adjusted model^2^	1.05 (0.12–1.98)	0.028	0.62 (−0.01–1.26)	0.055

1 β-coefficients for usual sodium and potassium intake represent the change in mmHg of blood pressure associated with 1,000 mg/d change in intake, whereas the β-coefficient for sodium-to-potassium ratio is per 0.5 unit change in intake.

2 Adjusted for age as categorical variable (in every 5-year increment), gender, race/ethnicity, body mass index, education, use of table salt, smoking status, history of cardiovascular disease, self-reported chronic kidney disease, diabetes mellitus, alcohol use and physical activity. Sodium and potassium intake were adjusted for concurrently in the same model, and the models for sodium-to-potassium ratio did not adjust for sodium and potassium intake.

**Figure 2:**
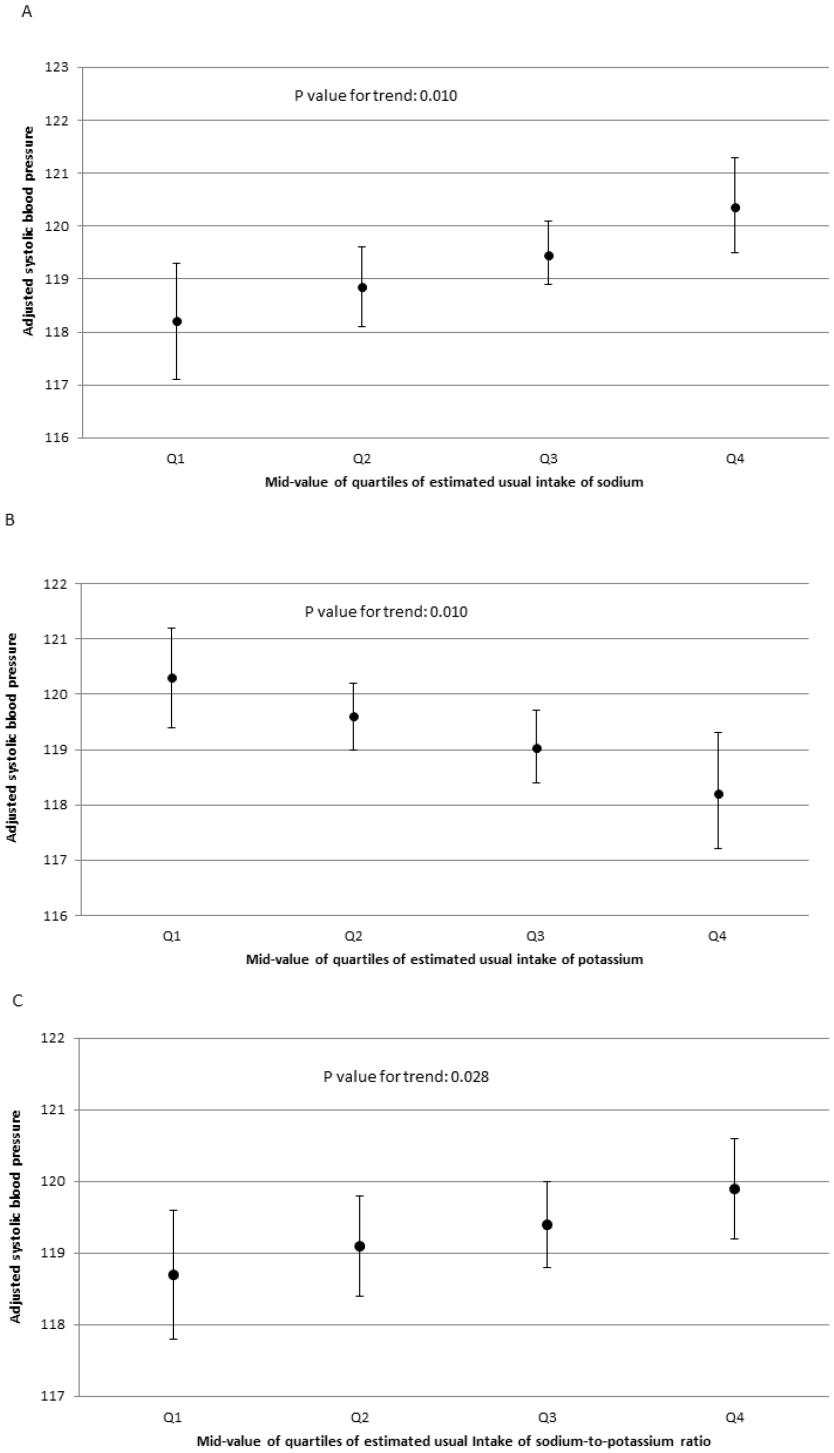
Adjusted systolic blood pressure (95% confidence interval) by mid-value of quartile sodium, potassium intake and their ratio among adults aged ≥20 years who were not taking antihypertensive medication, NHANES 2005–2010.

The adjusted average systolic blood pressure among study participants ranged from 118.2 mmHg (95% CI, 117.1–119.3) among those in the lowest quartile of sodium intake to 120.4 mmHg (95% CI, 119.5–121.3) (p = 0.010) among those in the highest (Figure 2A); from 120.3 mmHg (95% CI, 119.4–121.2) among those in the lowest quartile of potassium intake to 118.2 mmHg (95% CI, 117.2–119.6) (p = 0.010) among those in the highest (Figure 2B); and from 118.7 mmHg (95% CI, 117.8–119.6) among those in the lowest quartile of sodium-to-potassium ratio to 119.9 mmHg (95% CI, 119.2–120.6) among those in the highest (p = 0.028) (Figure 2C).

The adjusted ORs comparing prevalence of hypertension among adults in the highest quartile vs. those in the lowest quartiles were 1.40 (95% CI, 1.07–1.83) for sodium intake, 0.72 (95% CI, 0.53–0.97) for potassium intake, and 1.30 (95% CI, 1.05–1.61) for sodium-to-potassium ratio (Table 4). Adjusted ORs were 1.16 (95% CI, 1.02–1.32), 0.81 (95% CI, 0.67–0.98) and 1.26 (95% CI, 1.05–1.51), respectively, for every 1,000 mg/d increase in sodium and potassium intake and every 0.5 unit increase in the sodium-to-potassium ratio. The associations did not differ significantly by age, gender, race/ethnicity, BMI, or education (P>0.06 for all interactions).

**Table 4:** Adjusted odds ratio (OR) of estimated usual sodium and potassium intake and their ratio for hypertension among adults aged ≥20 years who were not taking antihypertensive medication, NHANES 2005–2010.

	Mid-value of quartiles of estimated usual intake in population				
Characteristics	Q1	Q2	Q3	Q4	p-value for trend^1^
**Usual sodium intake (mg/d)**	2,540	3,165	3,837	4,767	
Adjusted for age, sex and race/ethnicity only	1.0	1.11 (1.03–1.20)	1.25 (1.07–1.46)	1.46 (1.12–1.91)	0.007
Fully-adjusted model^2^	1.0	1.10 (1.02–1.18)	1.21 (1.04–1.42)	1.40 (1.07–1.83)	0.020
**Usual potassium intake (mg/d)**	1,952	2,466	2,953	3,598	
Adjusted for age, sex and race/ethnicity only	1.0	0.86 (0.79–0.93)	0.74 (0.63–0.87)	0.61 (0.47–0.80)	<0.001
Fully-adjusted model^2^	1.0	0.90 (0.82–0.99)	0.82 (0.68–0.98)	0.72 (0.53–0.97)	0.027
**Sodium-to-potassium ratio**	1.11	1.31	1.47	1.69	
Adjusted for age, sex and race/ethnicity only	1.0	1.13 (1.05–1.20)	1.24 (1.10–1.40)	1.41 (1.16–1.71)	<0.001
Fully-adjusted model^2^	1.0	1.10 (1.02–1.18)	1.18 (1.03–1.35)	1.30 (1.05–1.61)	0.016

1 P-value for trend across percentiles of estimated usual intake of sodium based on Satterthwaite adjusted F-test; all tests were two-tailed.

2 Adjusted for age as categorical variable (in every 5-year increment), gender, race/ethnicity, body mass index, education, use of table salt, smoking status, history of cardiovascular disease, self-reported chronic kidney disease, diabetes mellitus, alcohol use and physical activity.

Sodium and potassium intake were adjusted for concurrently in the same model, and the models for sodium-to-potassium ratio did not adjust for sodium and potassium intake.

In a sensitivity analysis, we used intake data from the 1^st^ 24-hour dietary recall to examine their association with blood pressure and hypertension, and found that compared with the results using the usual intake, the mean intake of sodium, potassium and their ratio from the 1^st^ 24-hour dietary recall did not change significantly (Table S1 in File S1), but substantial attenuation occurred in the association between all three intake variables and blood pressure and hypertension (Tables S2 and S3 in File S1).

We repeated analyses including 3,780 NHANES participants who took antihypertensive medications. The results of this analysis generally showed a greater correlation between the three intake variables and blood pressure than did the results of our main analyses in which we excluded participants who took antihypertensive medications (Tables S4 and S5 in File S1).

## Discussion

In this large nationally representative sample of U.S. adults who were not taking anti-hypertensive medications, we found that usual sodium intake and sodium-to-potassium ratio were positively associated and usual potassium intake was negatively associated with blood pressure and hypertension. After adjustment for demographic and other characteristics, the pattern of association was largely consistent across gender, race/ethnicity, education, and BMI categories. However, the effect of potassium intake on the systolic blood pressure appeared to be stronger among the older than among younger participants (p<0.001 for interaction).

Our findings of positive association between sodium intake and blood pressure were consistent with results from animal experiments, epidemiological studies, and clinical trials [8], [29]–[35]. A recent meta-analysis of 34 trials reported that a 75 mmol per 24 hours reduction in urinary sodium excretion (equivalent to sodium reduction of 1760 mg/d) reduced on average −4.18 mmHg (95% CI, −5.18, −3.18) for systolic and −2.06 mmHg (95% CI, −2.67, −1.45) for diastolic blood pressure [36].

Although we found potassium intake to be negatively associated with blood pressure and hypertension, results from previous studies of this relationship have been inconsistent. Our results are difficult to compare with these previous studies because of differences in the characteristics of study participants [8]–[14], or in methods used to assess potassium intake [26]. Of three studies whose results did not show an association between potassium intake and systolic blood pressure [12]–[14], one assessed participants' potassium intake on the basis of a single 24-hour dietary recall [12]; the lack of association may thus possibly be related to measurement error caused by day-to-day variability in potassium intake as suggested by the attenuation of our results when we used only one 24-hour diet recall. The three other studies adjusted for energy intake among other variables [11], [13], [14]. However, had we adjusted for energy intake in our study using the residual method, the estimated decrease in systolic blood pressure associated with a 1,000 mg/d increase in potassium intake would still have been significant (1.86 mmHg (95% CI, 0.92–2.80)) for every 1,000 mg/d increase in potassium intake. The InterSalt study used one 24-hour potassium excretion and found that potassium excretion was negatively correlated with both systolic and diastolic blood pressure in individual subjects after adjustments for confounding variables such as age, gender, BMI, alcohol intake, and sodium excretion [35]. A recent meta-analysis of 21 randomized controlled trials reported that increased 24 hour urinary potassium excretion reduced systolic blood pressure by 5.93 mmHg (95% CI, 1.70, 10.15) and diastolic blood pressure by 3.78 mmHg (95% CI, 1.43, 6.13) based on the mean differences between intervention and control groups [37]. In addition, we found a significant interaction between potassium intake and age on the systolic blood pressure. The stratified analysis suggested that the effect of potassium intake on systolic blood pressure appeared to be stronger among older age groups. However, the possible biologic mechanism of differential effects of potassium intake on blood pressure by age is unknown, and we cannot rule out the possibility that the significance of these findings are by chance. Further study is needed to clarify this association.

Two studies examined the relationship between blood pressure and sodium-to-potassium ratio from dietary recall data [16], [17]. Both studies used intake estimated derived from a single 24-hour recall, and neither adjusted for chronic kidney disease, diabetes, alcohol use, or physical activity. However, the results of both showed a positive associations between subjects' blood pressure and their sodium-to-potassium ratio, but the association was found only among people who consumed <400 mg of dietary calcium intake per day in one study [33]. In these two studies, the magnitudes of the increase in systolic blood pressure per unit change in sodium-to-potassium ratio (2.2 and 2.8 mm Hg) was similar to our finding of a 2.1 mm Hg increase. In the sensitivity analyses, we found no significant difference in the magnitude of association by levels of usual dietary calcium intake (<400 mg/d, 400–800 mg/d and >800 mg/d) (results not shown). The InterSalt study found that 24-hour urinary excretion of sodium-to-potassium ratio was also positively related to blood pressure after adjustments for age, gender, BMI, and alcohol intake [35]. Similar results were found from the Dallas Heart Study using urinary excretion of sodium-to-potassium ratio after adjustments for age, gender, race, diabetes, BMI, total cholesterol, estimated glomerular filtration rate, and urine albumin/creatinine ratio [38].

According to the World Health Organization, the sodium-to-potassium ratio should be ≤1 [39] which is also consistent with the Dietary Guidelines for Americans [40]. In the Dietary Approaches to Stop Hypertension (DASH) diet, which is rich in potassium-containing fruits, vegetables, and low-fat dairy foods, the target for sodium and potassium consumption were 3,000 mg and 4,700 mg (0.6 sodium-to-potassium ratio) for 2,100 kcal diet per day, respectively [41]. This DASH diet was shown to be effective in decreasing blood pressure levels in adults without hypertension and adults with stage I isolated systolic hypertension. Further decreases in average sodium intake (to 2,300 and 1,500 mg/d) and sodium-to-potassium ratio (to 0.5-0.3) provided additional benefits. Results from other studies have shown that a low-sodium/high-potassium diet also decreases blood pressure in adults on antihypertensive medications [42], [43]. In one of these studies, participants' sodium intake was lowered by providing them with sodium-free bread [42]. Given that grain products are a major source of sodium in the American diet [44], the increased availability of low-sodium products coupled with increased availability of low-cost fruits and vegetables may make it easier for U.S. adults to adopt low-sodium/high-potassium diets.

Our study has major strengths. This is, to our knowledge, the first study to assess the association between usual intake, particularly sodium-to-potassium ratio, and blood pressure and hypertension status in large nationally representative sample of U.S. adults. We used a measurement error model to estimate usual sodium, potassium intake and their ratio from two 24-hour dietary recalls accounting for within individual variation in intake. We also examined interactions with important sociodemographic and clinical characteristics.

A number of issues must be taken into account when interpreting our results. First, our reliance was upon results of 24-hour dietary recall to estimate participants' usual sodium and potassium intake. Compared with 24-hour sodium excretion, sodium intake from repeated 24-hour dietary recall interviews in NHANES may under or overestimate actual intake. Sodium intake is highly correlated with energy intake and a previous validation study using the 24-hour dietary recall used in this study suggests energy intake from 24-hour dietary recalls may underestimate energy intake by 11% [45]. However, both sodium and potassium intake estimated from replicate 24-hour dietary recalls correlates significantly with 24-hour excretion and studies suggest data from recalls can provide a valid estimate of associations [46], [47]. Second, our estimation of usual sodium and potassium intakes excluded sodium and potassium from table salt, supplements and antacids, resulting in an underestimate of overall sodium and potassium intake. However, the contributions of these additional components to overall sodium and potassium intake are small (<6% all together). Third, the clinical classification of hypertension among individuals is based on the average of two seated blood pressure measurements, properly measured with well-maintained equipment, at each of two visits to the office or clinic (21). As blood pressure in this study was measured during a single mobile examination visit, some individuals without hypertension may be included among those with hypertension, likely diminishing the associations. Fourth, questions for physical activity differed between the NHANES 2005–2006 and 2007–2010 cycles. To minimize the influence of this inconsistency in physical activity measure, we defined less than 10 minutes of moderate and/or vigorous activity per week as physically inactive. Furthermore, our study was cross-sectional, thus the associations between sodium, potassium and their ratio and blood pressure and hypertension should be interpreted with caution. Finally, we cannot rule out unmeasured confounding factors. For example, there might be unmeasured medical conditions that might explain the association between sodium, potassium intake and their ratio with blood pressure and the hypertension.

Our results indicated that sodium intake was positively associated with systolic blood pressure and hypertension, that potassium intake was negatively associated with both, and that the sodium-to-potassium ratio was positively associated with both. These results support those from randomized controlled trials showing reduced sodium consumption and increased potassium consumption can help prevent hypertension, and hence, cardiovascular disease.

## Supporting Information

File S1 **Table S1, Average intake of sodium, potassium and sodium-to-potassium ratio from the 1^st^ 24-hour dietary recall among U.S. adults aged ≥20 years who were not taking antihypertensive medication, by hypertension status selected characteristics, NHANES 2005–2010. Table S2, Association between intake of sodium, potassium and their ratio from the 1^st^ 24-hour dietary recall and blood pressure among U.S. adults aged ≥20 years who were not taking antihypertensive medication, NHANES 2005–2010. Table S3, Adjusted odds ratio (OR) of sodium and potassium intake from the 1^st^ 24-hour dietary recall and their ratio for hypertension among U.S. adults aged ≥20 years who were not taking antihypertensive medication, NHANES 2005–2010. Table S4, Association between usual intake of sodium, potassium and their ratio and blood pressure among U.S. adults aged ≥20 years, NHANES 2005–2010. Table S5, Adjusted odds ratio (OR) of estimated usual intake of sodium, potassium and their ratio for hypertension among U.S. adults aged ≥20 years, NHANES 2005–2010.**
(DOCX)
