# Supplementary material for: Association between Usual Sodium and Potassium Intake and Blood Pressure and Hypertension among U.S. Adults: NHANES 2005–2010
Source: PLoS One. 2013 Oct 10;8(10):e75289. doi: 10.1371/journal.pone.0075289 (PMC3794974; doi:10.1371/journal.pone.0075289)
Supplement: File S1 — Table S1, Average intake of sodium, potassium and sodium-to-potassium ratio from the 1st 24-hour dietary recall among U.S. adults aged ≥20 years who were not taking antihypertensive medication, by hypertension status selected characteristics, NHANES 2005–2010. Table S2, Association between intake of sodium, potassium and their ratio from the 1st 24-hour dietary recall and blood pressure among U.S. adults aged ≥20 years who were not taking antihypertensive medication, NHANES 2005–2010. Table S3, Adjusted odds ratio (OR) of sodium and potassium intake from the 1st 24-hour dietary recall and their ratio for hypertension among U.S. adults aged ≥20 years who were not taking antihypertensive medication, NHANES 2005–2010. Table S4, Association between usual intake of sodium, potassium and their ratio and blood pressure among U.S. adults aged ≥20 years, NHANES 2005–2010. Table S5, Adjusted odds ratio (OR) of estimated usual intake of sodium, potassium and their ratio for hypertension among U.S. adults aged ≥20 years, NHANES 2005–2010. (DOCX) [file pone.0075289.s001.docx]

**Table S1 Average intake of sodium, potassium and sodium-to-potassium ratio from the 1^st^ 24-hour dietary recall among U.S. adults aged ≥20 years who were not taking antihypertensive medication, by hypertension status selected characteristics, NHANES 2005-2010^1,2^**

| **Category** | **Sample N (%)** | | **Usual sodium intake, mean (SE), mg/d** | | **Usual potassium intake, mean (SE), mg/d** | | **Sodium-to-potassium ratio, mean (SE)** | |
| --- | --- | --- | --- | --- | --- | --- | --- | --- |
|  | **Hypertensive** | | **Hypertensive** | | **Hypertensive** | | **Hypertensive** | |
|  | **Yes** | **No** | **Yes** | **No** | **Yes** | **No** | **Yes** | **No** |
| **All** | 2178 | 7353 | 3623 (51.5) | 3613 (31.3) | 2795 (45.5) | 2757 (24.4) | 1.41 (0.02) | 1.43 (0.02) |
| **Age, years** |  |  |  |  |  |  |  |  |
| 20-50 | 969 | 5998 | 3973 (87.6) | 3723 (35.0) | 2869 (65.3) | 2722 (29.7) | 1.53 (0.03) | 1.50 (0.02) |
| ≥51 | 1209 | 2387 | 3248 (76.5) | 3286 (44.1) | 2716 (51.6) | 2861 (40.1) | 1.27 (0.02) | 1.22 (0.02) |
| **Sex** |  |  |  |  |  |  |  |  |
| Male | 1231 | 4202 | 4226 (71.7) | 4244 (45.6) | 3183 (59.6) | 3149 (33.7) | 1.42 (0.02) | 1.45 (0.01) |
| Female | 947 | 4183 | 2842 (52.8) | 3019 (32.0) | 2292 (57.1) | 2389 (25.3) | 1.39 (0.04) | 1.39 (0.03) |
| **Race/ethnicity** |  |  |  |  |  |  |  |  |
| Non-Hispanic white | 1055 | 4003 | 3693 (70.2) | 3664 (38.1) | 2901 (55.1) | 2838 (31.8) | 1.37 (0.02) | 1.41 (0.02) |
| Non-Hispanic black | 470 | 1403 | 3386 (120.2) | 3484 (67.4) | 2354 (86.1) | 2352 (41.9) | 1.59 (0.05) | 1.61 (0.03) |
| Mexican-American | 396 | 1793 | 3155 (197.3) | 3396 (66.3) | 2673 (118.4) | 2694 (37.5) | 1.25 (0.05) | 1.36 (0.02) |
| Other | 257 | 1186 | 3810 (189.1) | 3591 (68.4) | 2686 (93.4) | 2650 (46.6) | 1.53 (0.07) | 1.47 (0.02) |
| **Body mass index** |  |  |  |  |  |  |  |  |
| <25.0 | 545 | 2945 | 3451 (105.4) | 3582 (51.3) | 2795 (102.4) | 2763 (43.2) | 1.36 (0.05) | 1.42 (0.04) |
| 25.0-29.9 | 744 | 2929 | 3583 (87.9) | 3614 (46.7) | 2836 (66.3) | 2861 (30.6) | 1.36 (0.03) | 1.37 (0.02) |
| ≥30.0 | 868 | 2466 | 3805 (83.8) | 3668 (50.3) | 2778 (65.6) | 2622 (32.1) | 1.48 (0.04) | 1.51 (0.02) |
| **History of cardiovascular disease^3^** |  |  |  |  |  |  |  |  |
| Yes | 212 | 334 | 3169 (153.8) | 3241 (153.4) | 2471 (127.6) | 2607 (122.4) | 1.44 (0.06) | 1.38 (0.06) |
| No | 1947 | 8028 | 3670 (56.5) | 3625 (31.4) | 2826 (52.4) | 2762 (23.7) | 1.40 (0.02) | 1.43 (0.02) |
| **Diabetes^4^** |  |  |  |  |  |  |  |  |
| Yes | 215 | 426 | 3122 (116.5) | 3637 (142.9) | 2449 (94.3) | 2844 (97.0) | 1.38 (0.05) | 1.37 (0.04) |
| No | 1962 | 7953 | 3658 (52.8) | 3612 (31.6) | 2819 (47.0) | 2754 (24.4) | 1.41 (0.02) | 1.43 (0.02) |
| **Self-reported chronic kidney disease^5^** |  |  |  |  |  |  |  |  |
| Yes | 63 | 97 | 2939 (229.0) | 3193 (337.2) | 2162 (170.3) | 2429 (281.0) | 1.46 (0.10) | 2.91 (1.36) |
| No | 2110 | 8276 | 3636 (52.6) | 3617 (31.4) | 2806 (46.9) | 2760 (24.5) | 1.41 (0.02) | 1.41 (0.01) |
| **Smoking status** |  |  |  |  |  |  |  |  |
| Current smoker | 562 | 2056 | 3632 (97.0) | 3690 (71.8) | 2768 (76.1) | 2713 (43.9) | 1.45 (0.04) | 1.50 (0.02) |
| Former smoker | 553 | 1727 | 3791 (131.6) | 3714 (66.0) | 2969 (79.2) | 2921 (46.0) | 1.35 (0.03) | 1.36 (0.03) |
| Never smoked | 1061 | 4601 | 3531 (105.9) | 3538 (39.1) | 2721 (83.1) | 2714 (32.2) | 1.41 (0.03) | 1.42 (0.03) |
| **Heavy user of alcohol^6^** |  |  |  |  |  |  |  |  |
| Yes | 343 | 1244 | 3852 (126.1) | 3708 (66.9) | 2981 (107.2) | 2807 (40.6) | 1.41 (0.04) | 1.41 (0.02) |
| No | 1722 | 6663 | 3583 (73.0) | 3612 (29.9) | 2754 (56.4) | 2758 (25.4) | 1.41 (0.03) | 1.43 (0.02) |
| **Physical activity^7^** |  |  |  |  |  |  |  |  |
| Active | 985 | 4552 | 3892 (77.2) | 3682 (38.1) | 3040 (70.0) | 2831 (29.0) | 1.36 (0.03) | 1.42 (0.04) |
| Inactive | 1169 | 3790 | 3335 (64.2) | 3502 (51.0) | 2529 (46.2) | 2636 (30.1) | 1.44 (0.03) | 1.43 (0.02) |

^1^.Sample size is unweighted. Pregnant women and individuals missing data on blood pressure measurement and hypertension status, reporting being on a low salt or low sodium diet or taking antihypertensive medication are excluded.

^2^.Hypertension included both diagnosed and undiagnosed hypertension. Participants were classified as having diagnosed hypertension if they indicated that a health care provider told them they had high blood pressure and as having undiagnosed hypertension if they indicated they had not been told they had high blood pressure but were found to have a mean systolic blood pressure ≥140 mmHg or a mean diastolic blood pressure ≥90 mmHg.

^3^.Cardiovascular diseases included self-reported history of coronary heart disease, heart attack, angina, chronic heart failure, or stroke.

^4^.Diabetes mellitus was based on participants’ self-reported history of diabetes diagnosis, or use of insulin or other diabetic medications to lower blood glucose;

^5^.Chronic kidney disease status was based on whether participants indicated they had “weak/failing kidneys”;

^6^.Heavy user of alcohol was defined as self-reported consumption of more than two beverages per day for men and more than one beverage per day for women;

^7^.Adults were classified as physically inactive if participants reported engaging in less than 10 minutes of moderate and/or vigorous-intensity activity per week.

**Table S2 Association between intake of sodium, potassium and their ratio from the 1^st^ 24-hour dietary recall and blood pressure among U.S. adults aged ≥20 years who were not taking antihypertensive medication, NHANES 2005-2010^1^**

|  | **Systolic** | | **Diastolic** | |
| --- | --- | --- | --- | --- |
| **Characteristics** | **β-coefficient^2^** | **p-value** | **β-coefficient^2^** | **p-value** |
| 1^st^ day sodium intake |  |  |  |  |
| Adjusted for age, sex and race/ethnicity only | 0.41 (0.12-0.69) | 0.006 | 0.19 (-0.003-0.39) | 0.053 |
| Fully-adjusted model^3^ | 0.25 (-0.05-0.55) | 0.102 | 0.10 (-0.10-0.30) | 0.325 |
| Usual potassium intake |  |  |  |  |
| Adjusted for age, sex and race/ethnicity only | -0.60 (-0.96--0.25) | 0.001 | -0.31 (-0.57--0.06) | 0.018 |
| Fully-adjusted model^3^ | -0.34 (-0.73-0.04) | 0.078 | -0.19 (-0.48-0.09) | 0.178 |
| Sodium potassium ratio |  |  |  |  |
| Adjusted for age, sex and race/ethnicity only | 0.22 (-0.14-0.59) | 0.222 | 0.11 (-0.06-0.29) | 0.195 |
| Fully-adjusted model^3^ | 0.14 (-0.12-0.41) | 0.278 | 0.10 (-0.03-0.23) | 0.144 |

^1^. Participants who reported taking anti-hypertensive medication (n=3,780) were excluded from the analyses.

^2^. The β-coefficients for sodium and potassium intake represent the change in mmHg of blood pressure associated with 1,000 mg/d change in intake, whereas the β-coefficient for sodium-to-potassium ratio is per 0.5 unit change in intake.

^3^. Adjusted for age as categorical variable (in every 5-year increment), gender, race/ethnicity, body mass index, education, use of table salt, smoking status, history of cardiovascular disease, self-reported chronic kidney disease, diabetes mellitus, alcohol use and physical activity. Sodium and potassium intake were adjusted for concurrently in the same model, and the models for sodium-to-potassium ratio did not adjust for sodium and potassium intake.

**Table S3** **Adjusted odds ratio (OR) of sodium and potassium intake from the 1^st^ 24-hour dietary recall and their ratio for hypertension among U.S. adults aged ≥20 years who were not taking antihypertensive medication, NHANES 2005-2010**^1^

|  | **Mid-value of quartiles of estimated usual intake in population** | | | | | |  | |  |
| --- | --- | --- | --- | --- | --- | --- | --- | --- | --- |
| **Characteristics** | **Q1** | | **Q2** | **Q3** | **Q4** | **p-value for trend**^2^ | | **Range of usual intakes/OR**^3^ | |
| Usual sodium intake/Range | 1,780 | | 2,793 | 3,830 | 5,563 |  | | 0-21,248 | |
| Adjusted for age, sex and race/ethnicity only | 1.0 | | 1.05 (1.01-1.10) | 1.11 (1.02-1.20) | 1.21 (1.04-1.41) | 0.018 | | 1.05 (1.01-1.10) | |
| Fully-adjusted model^4^ | 1.0 | | 1.04 (1.00-1.08) | 1.09 (1.00-1.18) | 1.16 (1.00-1.36) | 0.058 | | 1.05 (1.00-1.09) | |
| Usual potassium intake/Range | 1,428 | | 2,222 | 2,987 | 4,166 |  | | 4.5-14,666 | |
| Adjusted for age, sex and race/ethnicity only | 1.0 | | 0.92 (0.87-0.98) | 0.86 (0.75-0.97) | 0.76 (0.61-0.95) | 0.063 | | 0.93 (0.86-1.00) | |
| Fully-adjusted model^4^ | 1.0 | | 0.97 (0.85-1.00) | 0.86 (0.73-1.00) | 0.76 (0.58-1.00) | 0.400 | | 0.96 (0.88-1.05) | |
| Sodium-to-potassium ratio/Range | 0.78 | | 1.15 | 1.50 | 2.07 |  | | 0-36.444 | |
| Adjusted for age, sex and race/ethnicity only | 1.0 | 1.03 (1.00-1.06) | | 1.06 (1.00-1.12) | 1.11 (1.00-1.23) | 0.051 | | 1.04 (1.00-1.08) | |
| Fully-adjusted model^4^ | 1.0 | 1.02 (0.99-1.05) | | 1.05 (0.99-1.10) | 1.09 (0.99-1.19) | 0.106 | | 1.02 (0.99-1.06) | |

1. Participants who reported taking anti-hypertensive medication (n=3,780) were excluded from the analyses.
2. P-value for trend across percentiles of estimated usual intake of sodium based on Satterthwaite adjusted F-test; all tests two-tailed.
3. ORs are for an increase of 1,000 mg/d of intakes of sodium and potassium and an increase of per 0.5 unit of sodium-to-potassium ratio.
4. Adjusted for age as categorical variable (in every 5-year increment), gender, race/ethnicity, body mass index, education, use of table salt, smoking status, use of antihypertensive medication, history of cardiovascular disease, self-reported chronic kidney disease, diabetes mellitus, alcohol use and physical activity. Sodium and potassium intake were adjusted for concurrently in the same model, and the models for sodium-to-potassium ratio did not adjust for sodium and potassium intake.

**Table S4 Association between usual intake of sodium, potassium and their ratio and blood pressure among U.S. adults aged ≥20 years, NHANES 2005-2010^1^**

|  | **Systolic** | | **Diastolic** | |
| --- | --- | --- | --- | --- |
| **Characteristics** | **β-coefficient^2^** | **p-value** | **β-coefficient^2^** | **p-value** |
| **Usual sodium intake** |  |  |  |  |
| Adjusted for age, sex and race/ethnicity only | 1.80 (1.04-2.57) | <0.001 | 0.50 (0.006-0.98) | 0.047 |
| Fully-adjusted model^3^ | 1.36 (0.56-2.16) | 0.001 | 0.35 (-0.11-0.81) | 0.132 |
| **Usual potassium intake** |  |  |  |  |
| Adjusted for age, sex and race/ethnicity only | -1.95 (-2.81--1.08) | <0.001 | -0.54 (-1.04--0.05) | 0.033 |
| Fully-adjusted model^3^ | -1.39 (-2.34--0.44) | 0.005 | 0.55 (-1.00--0.01) | 0.019 |
| **Sodium-to-potassium ratio** |  |  |  |  |
| Adjusted for age, sex and race/ethnicity only | 1.86 (0.93-2.78) | <0.001 | 0.52 (-0.02-1.07) | 0.060 |
| Fully-adjusted model^3^ | 1.32 (0.34-2.30) | 0.001 | 0.48 (-0.05-1.01) | 0.075 |

^1^. Participants who reported taking anti-hypertensive medication (n=3,780) were included from the analyses.

^2^. The β-coefficients for usual sodium and potassium intake represent the change in mmHg of blood pressure associated with 1,000 mg/d change in intake, whereas the β-coefficient for sodium-to-potassium ratio is per 0.5 unit change in intake.

^3^. Adjusted for age as categorical variable (in every 5-year increment), sex, race/ethnicity, body mass index, education, use of table salt, smoking status, use of antihypertensive medication, history of cardiovascular disease, self-reported chronic kidney disease, diabetes mellitus, alcohol use and physical activity. Sodium and potassium intake were adjusted for concurrently in the same model, and the models for sodium-to-potassium ratio did not adjust for sodium and potassium intake.

**Table S5** **Adjusted odds ratio (OR) of estimated usual intake of sodium, potassium and their ratio for hypertension among U.S. adults aged ≥20 years, NHANES 2005-2010**^1^

|  | **Mid-value of quartiles of estimated usual intake in population** | | | | |  |  | |
| --- | --- | --- | --- | --- | --- | --- | --- | --- |
| **Characteristics** | **Q1** | **Q2** | **Q3** | **Q4** | **p-value for trend**^2^ | | **Range of usual intake/OR**^3^ | |
| **Overall population** |  |  |  |  |  | |  | |
| Usual sodium intake | 2,490 | 3,084 | 3,730 | 4,683 |  | | 782-7,933 |  |
| Adjusted for age, sex and race/ethnicity only | 1.0 | 1.15 (1.07-1.24) | 1.35 (1.16-1.56) | 1.69 (1.30-2.21) | <0.001 | | 1.27 (1.13-1.42) |  |
| Fully-adjusted model^4^ | 1.0 | 1.11 (1.03-1.19) | 1.23 (1.06-1.43) | 1.45 (1.11-1.89) | 0.005 | | 1.18 (1.05-1.33) |  |
| Usual potassium intake | 1,931 | 2,438 | 2,903 | 3,554 |  | | 741-7,464 |  |
| Adjusted for age, sex and race/ethnicity only | 1.0 | 0.82 (0.77-0.87) | 0.68 (0.60-0.76) | 0.52 (0.43-0.64) | <0.001 | | 0.69 (0.61-0.78) |  |
| Fully-adjusted model^4^ | 1.0 | 0.90 (0.84-0.97) | 0.82 (0.72-0.94) | 0.72 (0.58-0.91) | 0.004 | | 0.80 (0.69-0.92) |  |
| Sodium-to-potassium ratio | 1.10 | 1.29 | 1.45 | 1.67 |  | | 0.52-2.61 |  |
| Adjusted for age, sex and race/ethnicity only | 1.0 | 1.15 (1.08-1.21) | 1.28 (1.16-1.42) | 1.50 (1.27-1.78) | <0.001 | | 1.43 (1.23-1.66) |  |
| Fully-adjusted model^4^ | 1.0 | 1.09 (1.03-1.16) | 1.18 (1.05-1.32) | 1.31 (1.09-1.58) | 0.006 | | 1.27 (1.08-1.49) |  |

1. Participants who reported taking anti-hypertensive medication (n=3,780) were included from the analyses.
2. P-value for trend across percentiles of estimated usual intake of sodium based on Satterthwaite adjusted F-test; all tests were two-tailed.
3. ORs are for an increase of 1,000 mg/d of usual intake of sodium and potassium and an increase of per 0.5 unit of sodium-to-potassium ratio.
4. Adjusted for age as categorical variable (in every 5-year increment), sex, race/ethnicity, body mass index, education, use of table salt, smoking status, use of antihypertensive medication, history of cardiovascular disease, self-reported chronic kidney disease, diabetes mellitus, alcohol use and physical activity. Sodium and potassium intake were adjusted for concurrently in the same model, and the models for sodium-to-potassium ratio did not adjust for sodium and potassium intake.
